# Time spent outdoors in childhood related to myopia among young adults in the Swedish ABIS cohort

**DOI:** 10.1111/aos.16688

**Published:** 2024-04-09

**Authors:** Tomas Bro, Johnny Ludvigsson

**Affiliations:** ^1^ Department of Biomedical and Clinical Sciences Linköping University Linköping Sweden; ^2^ Division of Pediatrics, Department of Biomedical and Clinical Sciences Linköping University Linköping Sweden; ^3^ Center of Paediatrics and Gynaecology and Obstetrics, H.K.H. Kronprinsessan Victorias barn‐ och ungdomssjukhus. Region Östergötland Linköping Sweden

**Keywords:** ABIS, children, myopia, physical activity, prospective study, risk factors

## Abstract

**Purpose:**

Elucidate the prevalence of myopia among young adults from a birth cohort of Swedish children and its relationship to possible risk factors during their childhood.

**Methods:**

Five thousand two hundred young adults, mean 23.4 years and 58% females, participating in the prospective birth cohort All Babies in Southeast Sweden (ABIS) answered a questionnaire including questions regarding health and physical activity, spectacle use, myopia and age at first optical correction. Questionnaires at previous follow‐ups at ages 2–3, 5–6 and 8 years included information on type of housing, time outdoors, screen time and hours of reading. Myopia prevalence and associations with potential risk factors were analysed in univariate and multivariate regression models with Bonferroni's correction of *p*‐values.

**Results:**

In the ABIS Swedish birth cohort of young adults, the prevalence of myopia was 29%. A univariate logistic regression showed a higher odds ratio for myopia with female gender (OR 1.59; *p* < 0.05) and a completed and started university education (OR 1.52; *p* < 0.05). Significantly lower odds ratios were found for hours spent outdoors at 8 years of age (OR 0.82; *p* < 0.05). Multivariate logistic regression showed a higher odds ratio for myopia in females (OR 1.52–1.57; *p* < 0.05) and completed and started university education (OR 1.34–1.49; *p* < 0.05) in all models. In a model including accommodative effort, measured in diopter hours at 8 years of age, hours spent outdoors were associated with a lower odds ratio for myopia (OR 0.86; *p* < 0.05). No association could be detected between myopia and the type of housing or near work.

**Conclusion:**

The prevalence of myopia among young adults in a Swedish birth cohort was lower or unchanged compared to previous data. Female gender, higher education and less time spent outdoors in childhood were associated with an increased risk of developing myopia. Recommendations from child health services and schools should be given to stimulate children to spend enough time outdoors.

## 1 INTRODUCTION

Although high myopia carries the highest risk of complications, even low and moderate myopia increase the risk of cataract, glaucoma, retinal detachment and myopic macular degeneration (Haarman et al., 2020). According to the WHO, there has been an alarming increase in myopia over the last decades (WHO, 2017). The global prevalence is estimated to have increased from 23% to 28% between 2000 and 2010. However, there are considerable regional differences, with a prevalence of 5% in east Africa and 49% in high‐income Asia‐Pacific countries (Holden et al., 2016). However, the significance of the global increase is complicated to judge because of the lack of standardised definitions. Myopia is in addition more prevalent in younger and older individuals than in middle age, which needs to be taken into account when different studies are compared (Pan et al., 2015). Furthermore, the diagnosis is strongly dependent on whether examinations are performed in cycloplegia since myopia is defined as spherical equivalent (SE) ≤0.5 diopter has been shown to remain only in 53%–66% of cases after instillation of cycloplegic agents (Hu et al., 2015).

Several different factors are thought to lead to abnormal elongation of the eyes. A sibling to an identical twin with myopia has a 90% risk of developing the condition, and several genes linked to myopia have been identified (Kiefer et al., 2013). American children with two myopic parents had odds ratio (OR) 7.3 (95% CI 2.8–18.7) to develop myopia compared to children without parental myopia in a study including 366 individuals of 14 years age (Mutti et al., 2002). Data concerning the effect of gender is conflicting; females are shown to have both more and less myopia than males, indicating there is no simple correlation (Xiang & Zou, 2020). However, the rapid global increase cannot be explained solely by genetics; other factors such as environment and lifestyle must be considered. A meta‐analysis including 27 cohorts of children from all continents except South America showed that more time spent on near‐work activities was associated with higher odds of myopia (Huang et al., 2015). Time spent outdoors in daylight has also been presented as an independent protective factor. A systematic review showed that having a minimum of 2 h of outdoor activities can reduce the prevalence of myopia (Xiong et al., 2017). Type of housing might also be related to myopia; residing in a single‐family home was associated with a lower prevalence of myopia compared to an apartment among school‐aged children in China (Wu et al., 2016).

In the last decade, children have become users of digital devices at younger ages, with an increase in the use of computers, smart phones and tablets (Ofcom, 2022). In 2018, 78% of Swedish children had their own computer, compared to 56% in 2008 (Statistics Sweden, 2021). In 2017, 24% of Swedish children between 12 and 18 years of age had at least 3 h of daily screen time on weekdays and at least 10 h on weekends (Statistics Sweden, 2017). As screen time both increase near work and reduce time spent outdoors, it has been argued to play a role in myopia development. However, consistent evidence for this hypothesis is still lacking (Lanca & Saw, 2020). Beyond myopia as a dichotomic dependent variable, hours of schooling and outdoor exposure have also been related to the age of onset for myopia (Lanca et al., 2022).

Data on myopia among young adults (around 18–25 years of age) are limited. Data from Australia (2018–2022) have shown a prevalence of 26% in a 20‐year‐old population (Lee & Mackey, 2022), and data from Norway have shown a prevalence of 35% in a population aged 20–25 years (1996–1997) (Midelfart et al., 2002) and 17% in a population aged 17–19 years (2015–2016) (Hagen et al., 2018). Studies on Scandinavian male conscripts mean age 19 have reported myopia prevalence of 13% in Denmark (2004) (Jacobsen et al., 2007) and 22% in Finland around 2000 (Vannas et al., 2003).

Only a few myopia studies with Swedish data have been published in the last 20 years. Myopia that did not disappear when treated with Tropicamide, was found in 50% of about 1000 children aged 12–13 years in 1999 (Villarreal et al., 2000). Ten years later, 650 male conscripts between 17 and 23 years old were examined for refractive errors. Myopia was found in 38% but was not measured with cycloplegic refraction (Uhlin et al., 2009). A later and smaller study of 143 children aged 4–15 years found myopia in cycloplegic refraction at 6% (Gronlund et al., 2006). A more recent study found myopia (in cycloplegic refraction in the right eye) in 10% of 128 children aged 8–16 years old. Parental myopia was associated with both the level of myopia and the length of the eye (Demir et al., 2021).

The aim of this study was to elucidate the prevalence of myopia in a birth cohort of Swedish individuals and the myopia relationship to possible risk factors.

## 2 METHODS

All mothers who gave birth to a child between 1 October 1997 and 31 October 1999, in the Southeast of Sweden (*n* = 21 700) were asked to participate in the prospective cohort study All Babies in Southeast Sweden (ABIS) (Ludvigsson et al., 2001). The main purpose was to study the aetiology of autoimmune and other immune‐mediated diseases, including factors associated with the development of diabetes, such as physical activity. Parents were instructed to answer a comprehensive questionnaire regarding health, demographic data and psychosocial factors, including the parents' highest education and the living conditions of the child. Parents of 17 055 (78.6%) newborn children accepted to participate in ABIS. The study cohort was representative of Sweden with regard to parental educational level (Sepa et al., 2004).

Thereafter, questionnaires were completed at the birth of the child and then at regular check‐ups at 1, 2–3 and 5–6 years of age. At the age of 8 years, two questionnaires, one to a parent and one to the child, were sent home to the family and returned through mail. The number of daily hours the child spent on TV watching or computer/game activity was reported at 2–3, 5–6 and 8 years. Time spent outdoors was assessed at 2–3 and 8 years, and daily time spent reading was assessed at 8 years of age.

During 2021–2022, all individuals in ABIS at birth (at that time 22–24 years of age) were contacted by mail with a new questionnaire regarding health and physical activity but also spectacle use, myopia and age at first optical correction. Self‐reported refractive errors have been shown to be a valid method to measure myopia in previous studies (Breslin et al., 2014; Ip et al., 2007). All questions included in this study are presented as a supplement (Table S1). To evaluate if the 22–24‐years group was representative of the birth group, the two groups were compared by gender and mothers education at birth questionnaire.

This study focuses on the prevalence of myopia in the cohort of young adults in the ABIS study and the possible association between myopia and the possible risk factors: gender, type of housing, time outdoors, screen time and hours of reading. To measure the effect of near work not only in terms of time, but also in terms of accommodative effort, diopter hours (DH) were calculated according to previously described methods (Mutti et al., 2002). One DH was defined as 3 * hours of reading +2 * hours spent playing video games or working on the computer at home +1 * hours spent watching television.

A univariate regression analysis was performed to identify possible risk factors for myopia. Moreover, a multivariate regression analysis was performed to identify possible risk factors for myopia at different ages of questionnaire responses. Findings were reported as odds ratios with a 95% confidence interval and two‐tailed *p*‐values. The alpha level of *p* < 0.05 was always used. To adjust for type I errors, Bonferroni's correction was applied in all regressions. As the structure of the study sample made the number of cases vary between different methods of analysis, *n* was reported separately for each method. The ABIS study has ethical approvals from the Research Ethics Committees of the Faculty of Health Science at Linköping University, Sweden, Dnr. 1997/9628 and 2003/03‐092 and for the adult follow‐up Dnr 2019‐05227.

## 3 RESULTS

**FIGURE 1:**
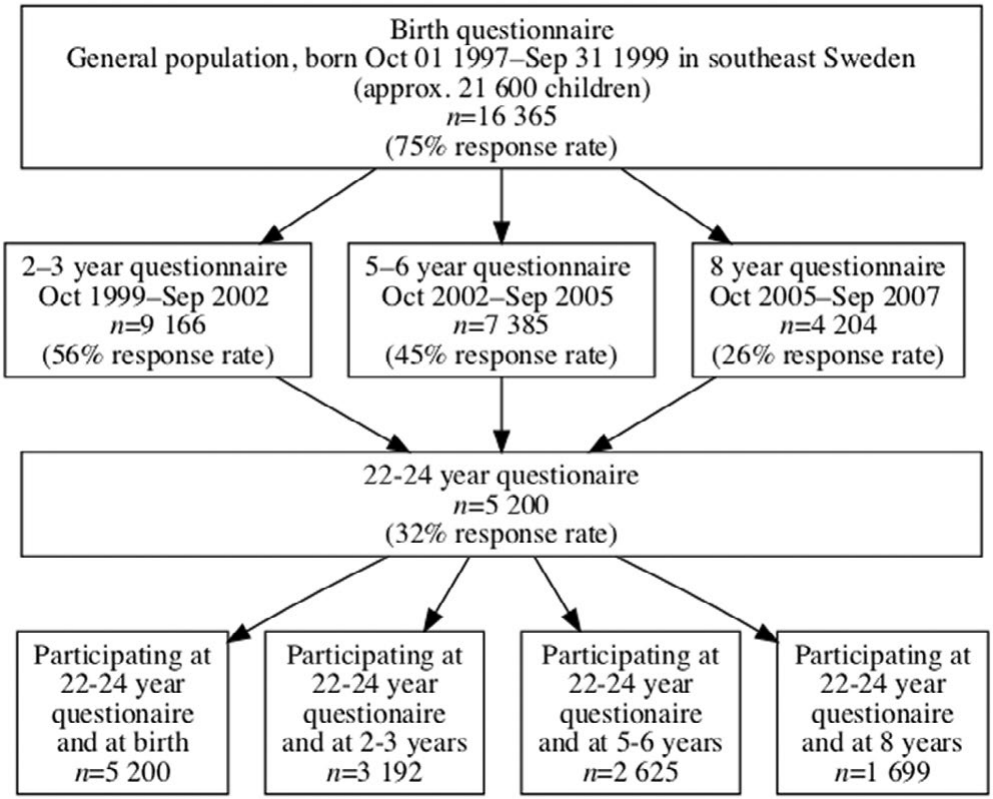
Flow diagram of the study sample.

**FIGURE 2:**
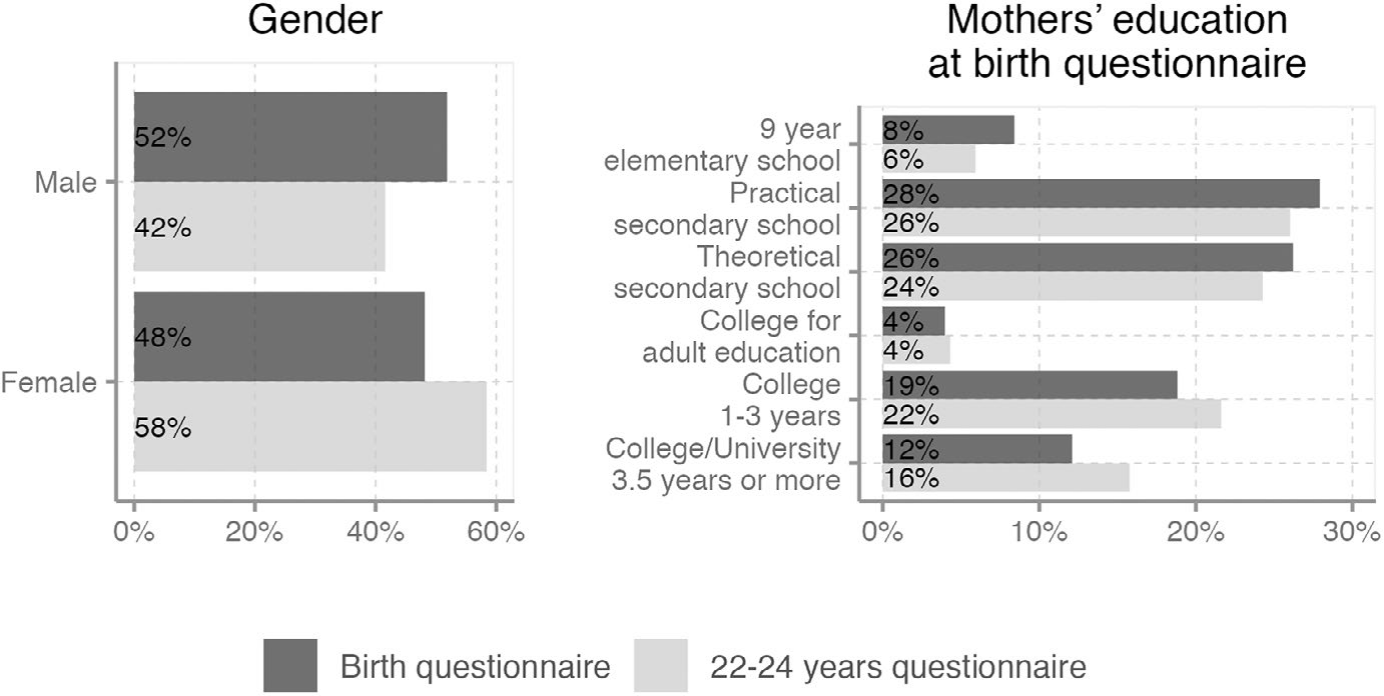
Distribution of gender and mothers education at birth questionnaire at the birth questionnaire and for the 22–24 years questionnaire.

The number of completed questionnaires at ages 22–24 was 5200 (Figure 1), whereof 58% were of female gender. The mean age was 23.4 and ranged from 22.6 to 24.0 (Table 1). Compared to the birth group, the 22–24‐year group had a higher proportion of female participants, and their mothers had higher education at the time of the birth questionnaire (Figure 2).

**TABLE 1:** Data characteristics responders to ABIS‐questionnaire at 22–25 years of age.

Factor	Value
*n*	5200
Mean age (min–max)	23.4 (22.6–24.0)
Female gender	58%
Any optical correction	41%
Optical correction for myopia	29%
Optical correction for myopia male gender	23%
Optical correction for myopia female gender	33%
Median age at first correction myopia (IQR)	15.0 (6.0)
Median age at first correction other (IQR)	16.0 (11.0)
Completed or started university education 22–24 years of age	55%
Parent with university education birth questionnaire	47%
Apartment instead of house 2.5 years of age	19%
Apartment instead of house 8 years of age	11%
Average hours outdoors 2.5 years of age	3.1
Average hours outdoors 8 years of age	2.7
Average hours with screens 2.5 years of age	1.8
Average hours with screens 5 years of age	2.7
Average hours with screens 8 years of age	2.5
Average hours reading 8 years of age	0.6
Average near work diopter hours 8 years of age	4.9

Abbreviation: IQR, interquartile range.

The prevalence of any optical correction was 41% and the prevalence of myopia was 29%. Myopia was more common among women (33%) than men (23%) (*p* < 0.05). The median age for first correction was lower for myopia (15) than for other refractive errors (16) (Table 1). The distribution of age for first correction was unimodal for myopia but bimodal for other refractive errors.

The number of available answers at 2–3, 5–6 and 8 years of age among the 5200 who answered questionnaires as adults varied between 1699 and 3192. A univariate logistic regression of possible risk factors for myopia showed a higher odds ratio for myopia with female gender (OR 1.59; *p* < 0.05) and a completed and started university education (OR 1.52; *p* < 0.05). A lower odds ratio was found for hours spent outdoors at 8 years age (OR 0.82; *p* < 0.05). None of the variables related to screen time or living conditions showed any significant association in this analysis (Table 2).

**TABLE 2:** Univariate regression of possible risk factors for myopia.

Factor (*n* available data)	OR (95% CI)	Bonferroni corrected *p*‐value
Female gender (4933)	1.59 (1.40–1.81)	<0.001
Parent with university education birth questionnaire (4842)	1.10 (0.97–1.24)	>0.9
Completed or started university education 22–24 years of age (4907)	1.52 (1.34–1.73)	<0.001
Apartment instead of house 2.5 years of age (2856)	0.98 (0.80–1.20)	>0.9
Apartment instead of house 8 years of age (1509)	1.35 (0.95–1.90)	>0.9
Average hours outdoors 2.5 years of age (2931)	0.93 (0.88–0.98)	0.10
Average hours outdoors 8 years of age (1558)	0.82 (0.74–0.91)	0.002
Average hours with screens 2.5 years of age (2847)	0.97 (0.90–1.05)	>0.9
Average hours with screens 5 years of age (2458)	0.96 (0.90–1.03)	>0.9
Average hours with screens 8 years of age (1571)	1.00 (0.90–1.10)	>0.9
Average hours reading 8 years of age (1574)	1.04 (0.78–1.36)	>0.9
Average diopter hours 8 years of age (1561)	1.00 (0.94–1.06)	>0.9

Abbreviations: CI, confidence interval; OR, odds ratio.

In multivariate logistic regression performed at different ages of questionnaire responses, a significantly higher odds ratio for myopia was shown for the female gender (OR 1.52–1.57; *p* < 0.05). In the models including data from 2.5 and 5 years of age, a significantly higher odds ratio for myopia was shown for completed and started university education (OR 1.34–1.49; *p* < 0.05). In a model including accommodative effort, measured in diopter hours at 8 years of age, hours spent outdoors were associated with a lower odds ratio for myopia (OR 0.86; *p* < 0.05). Parental education had no significant association in these models (Table 3).

**TABLE 3:** Multivariate regression of possible risk factors for myopia.

Model	Factor	OR (95% CI)	Bonferroni corrected *p*‐value
Model 1 (*n* = 2565)	Female gender	1.57 (1.31–1.88)	<0.001
	Parent with university education birth questionnaire	1.07 (0.89–1.28)	>0.9
	Apartment instead of house 2.5 of years	1.01 (0.81–1.25)	>0.9
	Average hours outdoors 2.5 years of age	0.94 (0.88–0.99)	0.2
	Average hours with screens 2.5 years of age	1.00 (0.92–1.08)	>0.9
	Completed or started university education 22–24 years of age	1.34 (1.11–1.61)	0.013
Model 2 (*n* = 2405)	Female gender	1.52 (1.26–1.85)	<0.001
	Parent with university education birth questionnaire	1.05 (0.87–1.26)	>0.9
	Average hours with screens 5 years of age	1.00 (0.93–1.07)	>0.9
	Completed or started university education 22–24 years of age	1.49 (1.23–1.80)	<0.001
Model 3 (*n =* 1396)	Female gender	1.57 (1.23–2.02)	0.003
	Parent with university education birth questionnaire	1.05 (0.82–1.34)	>0.9
	Apartment instead of house 8 years of age	1.40 (0.96–2.03)	0.5
	Average hours outdoors 8 years of age	0.86 (0.77–0.97)	0.076
	Average hours with screens 8 years of age	1.07 (0.95–1.20)	>0.9
	Average hours reading screens 8 years of age	0.98 (0.71–1.34)	>0.9
	Completed or started university education 22–24 years of age	1.39 (1.08–1.79)	0.080
Model 4 (*n =* 1396)	Female gender	1.55 (1.21–1.99)	0.003
	Parent with university education birth questionnaire	1.04 (0.81–1.32)	>0.9
	Apartment instead of house 8 years of age	1.40 (0.95–2.02)	0.5
	Average hours outdoors 8 years of age	0.86 (0.77–0.96)	0.036
	Completed or started university education 22–24 years of age	1.37 (1.07–1.77)	0.082
	Average diopter hours 8 years age	1.02 (0.96–1.09)	>0.9

Abbreviations: CI, confidence interval; OR, odds ratio.

## 4 DISCUSSION

This study of prevalence and possible risk factors for myopia showed a significantly lower odds ratio for myopia with hours spent outdoors at 8 years of age, but no significant association with screen time.

Although not fully representative of the general population, the prevalence of myopia among young adults in a Swedish birth cohort of 29% was lower or unchanged compared to previous data from Australia and Norway (Lee & Mackey, 2022; Midelfart et al., 2002). The prevalence of myopia of 22% among males is considerably lower than 38% among male Swedish conscripts age 17–23 in 2009 (Uhlin et al., 2009). That study defined myopia as a non‐cycloplegic autorefractor value as ≤−0.5 D, while we used self‐reported use of correction for myopia, which might explain this difference. Considering gender, females had a higher prevalence of myopia than males, which is in line with the majority of previous studies (Xiang & Zou, 2020). As the adult group had mothers with higher education at the time of the birth questionnaire compared to the birth group, it is not fully representative of the general population. Instead, the prevalence of myopia among young adults in Sweden might be even lower than 29%, as parental education has been shown as a risk factor in other previous research (Tang et al., 2020). With the limitations mentioned above, we cannot detect support for an increase in myopia at levels suggested by Holden et al. (2016) and adopted by WHO (2017).

More hours spent outdoors at 8 years of age had a significant association with lower odds of myopia, which is in line with previous data from Australia, Asia and UK (Xiong et al., 2017). No previous studies have used such a long follow‐up (14–16 years from the 8 ‐year questionnaire to follow‐up) and such a large cohort (5200). This result supports that early exposures to outdoor time have long‐term consequences. Increased time outdoors could therefore be used in policy programmes such as Tian Tian 120 in Taiwan and in other populations (Wu et al., 2020). No significant relationships could be detected between myopia and near work. However, screen time from 15 to 21 years ago meant TV and video watching, videogames or computers, all modalities that require less accommodative effort than contemporary tablets and smartphones (Mutti et al., 2002). No significant relationships were detected between the age of myopia onset and outdoor exposure or academic achievements, as shown in previous research (Lanca et al., 2022).

Even if parental education has been shown to increase the risk for myopia, our results suggest that the main factor is the inherited behaviour, as the association disappeared in models combined with the child's own educational achievements.

The strengths and limitations of this study are related to its design. It was a prospective, observational study following a large cohort of children from birth to 24 years of age in a non‐selected general population. As the study relied on self‐reports, factors such as screentime or outdoor time might be both over‐ and underreported, as no clear correlation between subjective and objective measures of near work and outdoor time has been observed in other studies (Dharani et al., 2012; Wen et al., 2021). However, there is no reason to expect any bias, as the data were registered many years before any questions about myopia were asked.

In summary, the prospective cohort study of a general population of individuals in Sweden found that female gender and less time spent outdoors in childhood increases the risk of developing myopia. Recommendations from child health services and schools should be given to stimulate children to spend enough time outdoors.

## FUNDING INFORMATION

This study was funded by Forte – Forskningsrådet för hälsa, arbetsliv och välfärd, (Grant number 2019‐00586). ABIS was supported by Barndiabetesfonden (Swedish Child Diabetes Foundation); Swedish Council for Working Life and Social Research, Grant/Award Numbers: FAS2004‐1775; Swedish Research Council, Grant/Award Numbers: K2005‐72X‐11242‐11A and K2008‐69X‐20826‐01‐4; Östgöta Brandstodsbolag; Medical Research Council of Southeast Sweden (FORSS); JDRF Wallenberg Foundation, Grant/Award Number: K 98‐99D‐12813‐01A; ALF‐and LFoU grants from Region Östergötland and Linköping university, Sweden; Joanna Cocozza Foundation.

## Supporting information


Table S1.
